# Advancing chemical safety assessment through an omics-based characterization of the test system-chemical interaction

**DOI:** 10.3389/ftox.2023.1294780

**Published:** 2023-11-09

**Authors:** Giusy del Giudice, Giorgia Migliaccio, Nicoletta D’Alessandro, Laura Aliisa Saarimäki, Marcella Torres Maia, Maria Emilia Annala, Jenni Leppänen, Lena Mӧbus, Alisa Pavel, Maaret Vaani, Anna Vallius, Laura Ylä‐Outinen, Dario Greco, Angela Serra

**Affiliations:** ^1^ Faculty of Medicine and Health Technology, Tampere University, Tampere, Finland; ^2^ Finnish Hub for Development and Validation of Integrated Approaches (FHAIVE), Tampere, Finland; ^3^ BioMediTech Unit, Tampere University, Tampere, Finland; ^4^ Division of Pharmaceutical Biosciences, Faculty of Pharmacy, University of Helsinki, Helsinki, Finland; ^5^ Institute of Biotechnology, University of Helsinki, Helsinki, Finland; ^6^ Tampere Institute for Advanced Study, Tampere University, Tampere, Finland

**Keywords:** chemical safety assessment, new approach methodologies, toxicogenomics, test system characterization, AOP, nanosafety, chemical-biological interaction, mechanism of action

## Abstract

Assessing chemical safety is essential to evaluate the potential risks of chemical exposure to human health and the environment. Traditional methods relying on animal testing are being replaced by 3R (reduction, refinement, and replacement) principle-based alternatives, mainly depending on *in vitro* test methods and the Adverse Outcome Pathway framework. However, these approaches often focus on the properties of the compound, missing the broader chemical-biological interaction perspective. Currently, the lack of comprehensive molecular characterization of the *in vitro* test system results in limited real-world representation and contextualization of the toxicological effect under study. Leveraging omics data strengthens the understanding of the responses of different biological systems, emphasizing holistic chemical-biological interactions when developing *in vitro* methods. Here, we discuss the relevance of meticulous test system characterization on two safety assessment relevant scenarios and how omics-based, data-driven approaches can improve the future generation of alternative methods.

## Introduction

Humans and the environment are exposed to various chemicals, generated from both human activities and natural sources, having the potential to cause detrimental effects. Chemical safety assessment aims at characterizing the possible hazards and risks associated with chemical exposures while also delineating the parameters under which substances can be safely used. Traditional approaches to chemical safety assessment rely on animal experimentation. However, with the rise of alternative methods rooted in the 3R principles (reduction, refinement, and replacement of *in vivo* experiments), *in silico* and *in vitro* approaches gained prominence (Burden et al., 2015; OECD, 2017; OECD, 2018a; Stucki et al., 2022).

The first phase of alternative approaches focuses on investigating specific toxicological endpoints by utilizing controlled *in vitro* environments. However, these methods do not provide mechanistic insights into chemical exposures. New Approach Methodologies (NAMs) instead, designed to mimic human biology, offer a deeper understanding of chemical-induced toxicity. Despite this advancement, NAMs still face a challenge in providing a structured framework for linking molecular events to adverse health and ecotoxicological outcomes. The Adverse Outcome Pathway (AOP) concept was introduced to address this gap. Adverse Outcome Pathways (AOPs) establish a coherent link between molecular initiating events (MIEs) and adverse outcomes (AOs), enhancing our understanding and prediction of toxicity mechanisms (OECD, 2017; Stucki et al., 2022).

The first generation of alternative approaches is mainly characterized by a chemocentric view. Indeed, their applicability domains define the validity of the tests for a group of substances with specific characteristics. However, as we navigate the landscape of alternative methods, it becomes increasingly evident that the nexus of chemical-biological interactions holds the key to developing more refined and effective models for chemical safety assessment. This is especially relevant for emerging classes of advanced materials such as nanomaterials, where the representation of the substance is not limited to the core characteristics but is affected by the external and environmental elements of the exposure (Wyrzykowska et al., 2022).

**FIGURE 1:**
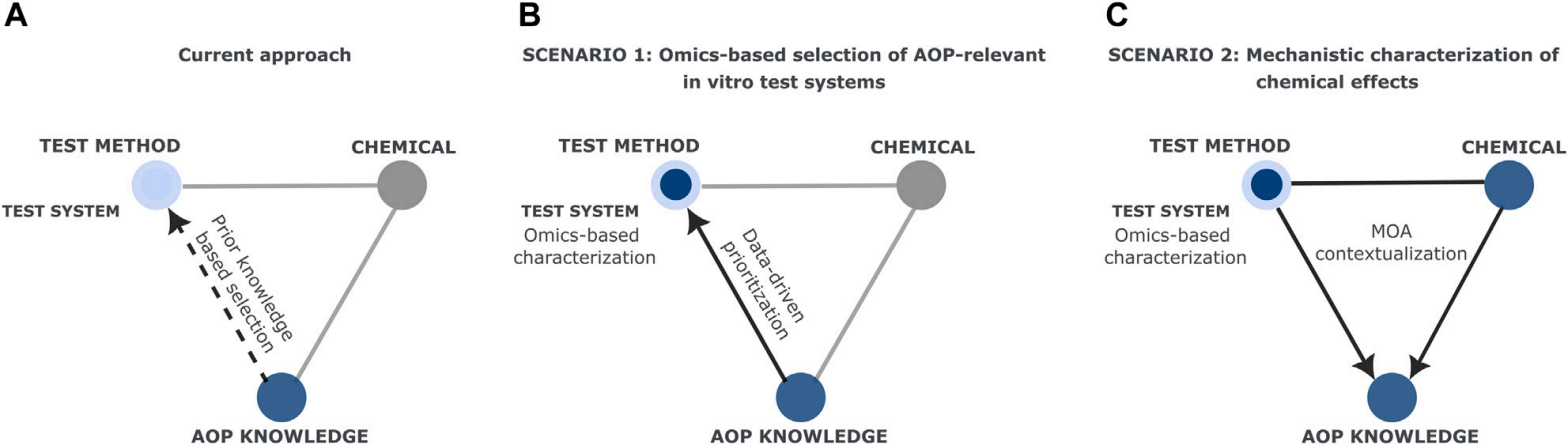
Scientific and regulatory scenarios based on the triangle of chemical safety from Carnesecchi *et al.* **(A)** Current selection of the *in vitro* test system mainly relies on prior knowledge from literature; **(B)** Suggested selection strategy of the test method including an omics-based data-driven selection of the *in vitro* test system; **(C)** Mechanistic characterization of chemical effects by a contextualized interpretation of omics data in *vitro* test system.

The relevance of *in vitro* test systems for studying chemical effects is predominantly determined based on their ability to mimic the phenotype of the target tissue, and practical considerations including availability, readiness, and ethical issues (Figure 1A) (Bruner et al., 1998; OECD, 2018a; Bernasconi et al., 2023). Preference often favors test systems due to their established use in toxicity testing or expression of specific markers (OECD, 2023). However, this decision is rarely based on how the complete molecular profile of the test system correlates with distinct physiological and pathological phenotypes *in vivo*, nor how it is relevant to specific toxicological endpoints. The absence of a comprehensive definition for the test system applicability domain currently impedes accurately describing the relationship between the biological system and the observed phenotype. To overcome this limitation, it is vital to meticulously characterize the variables of the test system, including the molecular machinery, to represent real-life exposure scenarios faithfully.

The introduction of mechanistic toxicology and toxicogenomics (TGx) has resulted in an increased understanding of how chemical substances induce their effects in biological systems (Serra et al., 2020a; Federico et al., 2020; Halappanavar et al., 2020; Kinaret et al., 2020; Serra et al., 2022a). Characterizing the mechanism of action of chemical exposures through TGx can support the identification of early molecular effects and predictive biomarkers for chemical safety assessment (Fortino et al., 2022). Similarly, TGx data can be used to derive points of departure through benchmark dose modelling and to support the definition of safe dose ranges (Labib et al., 2016; Serra et al., 2020b; Serra et al., 2020c; Saarimäki et al., 2020; Serra et al., 2022b). Finally, TGx analysis can support effective *in vitro* to *in vivo* extrapolation, hence guiding the development of NAMs (Kinaret et al., 2017; Saarimäki et al., 2023a). These examples highlight the potential of TGx in chemical safety assessment. The enormous amounts of data and resources generated by the TGx field can be exploited to develop robust chemical safety assessment methodologies with emphasis on the chemical-biological system interaction, expanding the current practices from a chemocentric view to an integrated, holistic approach.

Despite the concerns and limitations (Sauer et al., 2017; Gant et al., 2023) of integrating omics data in chemical safety assessment, regulatory agencies are actively exploring their mechanistic value using them as weight-of-evidence (Bridges et al., 2017; OECD, 2018b). Notably, the OECD has approved the first omics-based model for screening molecular biomarkers related to skin sensitization (Johansson et al., 2013; OECD, 2018c). Frameworks and strategies for standardization are emerging to facilitate their incorporation into regulatory-approved settings (van der Zalm et al., 2022; Bleeker et al., 2023). Hence, it is plausible to envision that by implementing standardized and reproducible methodologies, omics will become commonplace in future safety assessments, thereby enabling its implementation in defining the applicability domains of the test systems.

Recently, Carnesecchi et al. (Carnesecchi et al., 2023) introduced the notion of the triangle of chemical safety, highlighting the need for integration and cross-reference of chemical data, test method information, and AOP knowledge to foster chemical safety assessment. We believe that alternative methods would greatly benefit from a deep characterization of the test system via omics approaches. We use the triangle of chemical safety as the base to showcase two scenarios underscoring the paramount importance of meticulous test system characterization (Figures 1B, C).

## Scenario 1: Omics-based selection of AOP-relevant *in vitro* test systems

In a typical safety assessment scenario, the evaluation of chemicals often begins with their physicochemical characterization^1^ ^,^ ^2^. Evaluating the properties of the chemicals and previous toxicological evidence is crucial to identify potential risks. In this phase, hazard assessment is implemented through endpoint-specific information strategies, where relevant AOPs are selected, and *in vitro* tests from the OECD-approved list are performed for each key event (KE).

For example, a recent validation study for detecting thyroid disruptive chemicals selected multiple *in vitro* tests covering relevant mechanisms associated with thyroid hormone system disruption (Bernasconi et al., 2023). The selection of the test methods was based on various factors, including laboratory expertise, procedural readiness, method comprehensiveness, and requirements on the test system, such as human relevance and minimization of inter-species variation.

As pointed out in the study, the test system selection holds a pivotal role in the prioritization of candidate test methods. This was performed by a thorough literature review demonstrating the relevance of test systems and their specificity for the distinct mechanism within the thyroid signaling pathway under investigation. A similar approach was used by Chary et al. (Chary et al., 2018), where the establishment of test systems for assessing multiple steps in the respiratory sensitization AOP relies on the pre-existing insights gained from test systems documented in the scientific literature.

However, cell lines expressing different genes have the potential to respond differently to the same compound (De Wolf et al., 2016; Winckers et al., 2021). Thus, we suggest the inclusion of a data-driven approach (Figure 1B) in the current process of selection of the most toxicologically relevant test system. This would be based on a comprehensive characterization of the molecular makeup of the *in vitro* system via omics profiling, that could be matched with the selected Key Events (KEs), for example, by exploiting the recent curation and annotation of genes and biological systems to KEs and AOPs (Saarimäki et al., 2023a; Saarimäki et al., 2023b). Recently, Black et al. showed how investigating the basal gene expression profiles of cell lines can give insights into their suitability for toxicity testing (Black et al., 2023). The development of many resources covering molecular profiles of various biological systems (ENCODE Project Consortium, 2012; GTEx Consortium, 2013; Thul and Lindskog, 2018) makes it possible to envision the link between the AOP annotation and steady-state expression levels. Such a data-driven approach would reduce manual effort, time, and costs. Furthermore, it would represent a generalized approach that can be applied across diverse studies.

**FIGURE 2:**
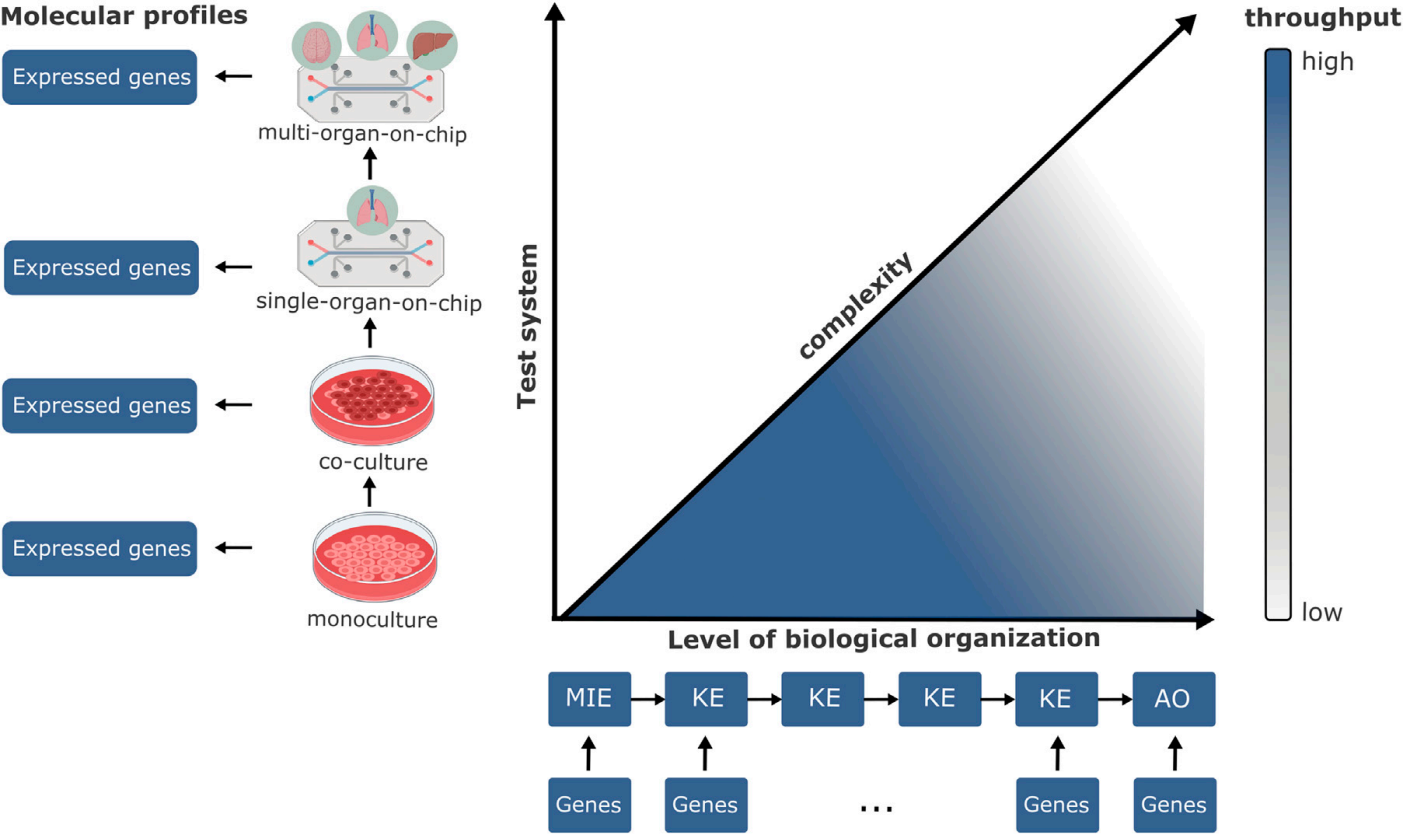
The relationship between the complexity of the test system and their relevance to key events (KEs) in the AOP framework. On the *x*-axis, a generic AOP structure is depicted, linking a direct molecular initiating event (MIE) to an adverse outcome (AO), at a level of biological organization relevant to hazard assessment (from cellular to population level). On the *y*-axis, test systems with increasing complexity are reported (from simple monoculture to multi-organ on-chip). Annotation of genes in the AOP framework allows the link between toxicologically relevant events and the molecular profiles derived from test systems.

It is important to note that the biological complexity of different test systems may affect their relevance for testing KEs at different levels of organization (Figure 2). According to the structure of the AOP, events closer to the Molecular Initiating Event (MIE) may be better represented and assessed in simpler *in vitro* tests based on individual biochemical assays within a monoculture experimental setup (e.g., ROS production). On the contrary, more apical events, closer to the Adverse Outcome (AO), may require a higher degree of organismal complexity, such as those provided by tissues or organs (e.g., lung fibrosis) (Noyes et al., 2019). In the context of the validation study focused on assessing *in vitro* techniques for identifying chemicals that influence thyroid function (Bernasconi et al., 2023), it becomes evident that higher complexity of the investigated KE corresponds to the utilization of more intricate test systems (e.g., co-culture and 3D models) resulting in a reduction in the number of tests created. On the other hand, when examining KEs closer to the MIE (e.g., TSH receptor activation, TPO inhibition), a broader array of options emerges for selecting both the test system and the method to implement (Bernasconi et al., 2023).

This complexity also affects the throughput of the test method to various chemicals. Early molecular events can be shared in the response of many compounds, therefore being the starting or intermediate steps toward multiple AOs. Ideally, high-throughput screening of a variety of compounds would be made in these settings. On the other hand, more complex test systems, such as organs-on-chips, may capture apical and specific endpoints very well (Ma et al., 2021), being tested for only limited sets of chemicals. However, they are subject to higher sources of variability (Leung et al., 2022) thus, many efforts are ongoing to improve their standardization (Mastrangeli and van den Eijnden-van Raaij, 2021; Piergiovanni et al., 2021). We believe that by providing a detailed understanding of the molecular and cellular responses within these systems through omics data characterization, a common reference point can be established. This systematic approach fosters a more robust foundation for collaborative research and the advancement of scientific knowledge in the field of chemical testing.

## Scenario 2: Advancing mechanistic characterization of chemical effects through contextualized interpretation of omics data in the joint test system-chemical applicability domain.

The distinct genetic characteristics of biological systems result in diverse responses to compounds, leading to heterogeneous effects and poor generalizability between *in vitro* and *in vivo* settings, and across different systems (Chang et al., 2021; Sachana et al., 2021; del Giudice et al., 2023). This variability impacts toxicity assessments, for example, requiring concurrent testing on rats and mice to account for potential over/underestimation of human toxicity (Saber et al., 2019).

When studying the effect of chemical exposure on a tissue or an organ, the result is the synergistic consequence of the responses exhibited by its constituent cellular populations, which cannot be simply modelled as their sum (Pognan et al., 2023). This phenomenon is the rationale behind the development of complex *in vitro* systems, where the co-culturing of principal cytological components of a tissue improves the precision of toxicological assessments (Pognan et al., 2023).

For example, Chortarea et al. described how co-culturing epithelial cells alongside dendritic cells and macrophages results in a more realistic exposure scenario when evaluating the effect of repeated exposure to carbon nanotube-based aerosols (Chortarea et al., 2015). Additionally, commercial sources provide airway liquid interface co-cultures, for instance, combining airway epithelial cells with fibroblasts (Hiemstra et al., 2018).

The influence of distinct cell populations is also evident in how AOPs are often developed. When formulating an *in vitro* battery of assays for developmental neurotoxicity assessment, Sachana et al. selected a variety of *in vitro* tests that span across neuronal populations to cover the landmarks of neurotoxicity (Sachana et al., 2021). The AOP is based on the principle that neuronal toxicity is the result of changes in various fundamental processes of neurodevelopment, and each of them needs to be tested on the relevant cell type. This process is common to many AOPs development strategies and has been previously applied to skin sensitization and endocrine disruption (Johansson et al., 2013; Bernasconi et al., 2023).

Therefore, it is clear that the biological system directly affects the observed response to compounds and that *in vitro* systems based on cell components of tissues often capture only a portion of the tissue/organ level toxicity. Although this effect is well known and is the conceptual basis of both co-culture systems and testing battery approaches, it is hardly considered when interpreting the mechanism of action of compounds, where omics alterations are usually interpreted from a chemocentric perspective.

Previous TGx analysis demonstrated how specific *in vitro* responses constitute components of the *in vivo* exposure effect, and therefore the observed cellular phenotype needs to be contextualized and characterized with respect to the cell system used (Kinaret et al., 2017). Similar considerations were reached when assessing the effects of MWCNT on the lungs and four cell lines (Saarimäki et al., 2023a). Comparing the *in vitro* responses to the *in vivo* counterpart revealed that cell lines easily show specific signals that are instead hidden in the *in vivo* system, where the co-existence of multiple cell types contributes to the observed phenotype.

Therefore, mechanistic interpretation of omics data requires contextualizing the observed outcomes in terms of the exposure and the exposed system. In our second scenario, we stress the importance of the biological system characterization when studying an exposure omics profile, where the chemical and the test systems are predefined (Figure 1C). This scenario applies both to cases in which the data has been newly generated, or when publicly available data are re-used for further toxicological assessment.

Our recent efforts demonstrated how to characterize gene alterations through robust bioinformatic pipelines that link molecular profiles to MIEs, KEs, and AOs for mechanistic insight (Saarimäki et al., 2023a; del Giudice et al., 2023). This comprehensive approach considers a broad range of potential AOs and prioritizes all appropriate biological responses that may lead to adverse outcomes. Similarly, Labib et al. suggested an integrated use of TGx data in the AOP framework (Labib et al., 2016).

We further envision that the mechanistic information retrieved from such approaches needs to be interpreted within the joint applicability domain of the test system and the chemical to unveil functional components of the real-life expected exposure response and lead to better characterization of the hazard potential and phenotype variability.

This approach addresses the challenge of utilizing omics data in regulatory settings, where their complexity and lack of straightforward interpretation have been limiting factors. Furthermore, by considering the impact of the biological system, it addresses the effect of biological variability, which has often hampered the comparison of individual exposures to the same compounds. Mapping the omics results to specific KEs within an AOP reduces the complexity of the interpretation (Saarimäki et al., 2023a; Saarimäki et al., 2023b). This enables a more manageable and meaningful understanding of the omics data, establishing a direct relationship with toxicologically relevant endpoints in regulatory assessment.

## Discussion

The field of toxicology is undergoing a significant shift towards alternative methods that aim to reduce reliance on animal experimentation (Burden et al., 2015), with AOPs and AOP-informed strategies playing a central role in this mechanistic understanding (Sakuratani et al., 2018).

However, most of the available alternative methods still lack a deep understanding of the chemical-biological interactions needed to advance the field of chemical safety assessment. While this is relevant for all chemicals, it holds particularly true for nanomaterials, where it has been proved that the potential hazard originates from the synergistic effect of the pristine characteristic and system-dependent elements of the surrounding environment (Wyrzykowska et al., 2022). This is of paramount importance when selecting the test method since different *in vitro* test systems will offer a specific or limited resemblance of the phenotypic effect *in vivo*. Thus, an omics-based thorough characterization of the *in vitro* test system is crucial for ensuring the reliability of the test method. It is imperative to evaluate if the test system expresses the appropriate molecular machinery relevant to the phenomena under study (Saber et al., 2019) and to determine if the effect of the chemical-biological interactions would correspond to real-life scenarios.

This could be achieved by enhancing the OECD-approved tests by incorporating a mechanistic layer that informs about the applicability domain of the biological test system. TGx data-driven analysis that prioritizes *in vitro* test systems for specific KEs would speed up the development of new alternative methods.

Similarly, the interpretation of the mechanism of action of chemical exposures should be contextualized with the biological system used to generate the molecular profile. This would not only allow a better understanding of the (partial) effect of the substance but also result in better prediction of phenotype variability and contribution to multiple adverse outcomes.

To stably implement the use of omics data derived information, the robustness and generalizability of *in vitro* assays and omics profiles must be ensured. Implementing Good Laboratory Practice (GLP) principles for omics data generation can boost transparency, reproducibility, and reliability while ensuring standardization of the experimental planning and reporting. (Kauffmann et al., 2017; Saarimäki et al., 2022).

Overall, a compelling necessity exists to strategically leverage the molecular machinery insight of *in vitro* test systems to prioritize them for diverse KEs. Such prioritization can delineate the biological applicability domain of the AOP, which encompasses a collection of *in vitro* systems viable for AOP testing. This knowledge could guide researchers in selecting the most pertinent system for their study and facilitating the design of more efficacious integrated test batteries.

By meticulously addressing these facets, we propel the advancement of NAMs and significantly streamline and enhance the accuracy of risk assessment within regulatory processes.

## Data Availability

The original contributions presented in the study are included in the article/Supplementary material, further inquiries can be directed to the corresponding author.

## Footnotes

1 European Chemical Agency. Guidance on Information Requirements and Chemical Safety Assessment. https://echa.europa.eu/guidance-documents/guidance-on-information-requirements-and-chemical-safety-assessment.

2 United States Environmental Protection Agency. Reviewing new Chemicals under the Toxic Substances Control Act (TSCA). https://www.epa.gov/reviewing-new-chemicals-under-toxic-substances-control-act-tsca.
